# Supplementary material for: The Effect of Storage Conditions on the Content of Molecules in Malus domestica ‘Chopin’ cv. and Their In Vitro Antioxidant Activity
Source: Molecules. 2022 Oct 17;27(20):6979. doi: 10.3390/molecules27206979 (PMC9610424; doi:10.3390/molecules27206979)
Supplement: Supplementary file 1 [file molecules-27-06979-s001.zip › molecules-1933660-supplementary.pdf]

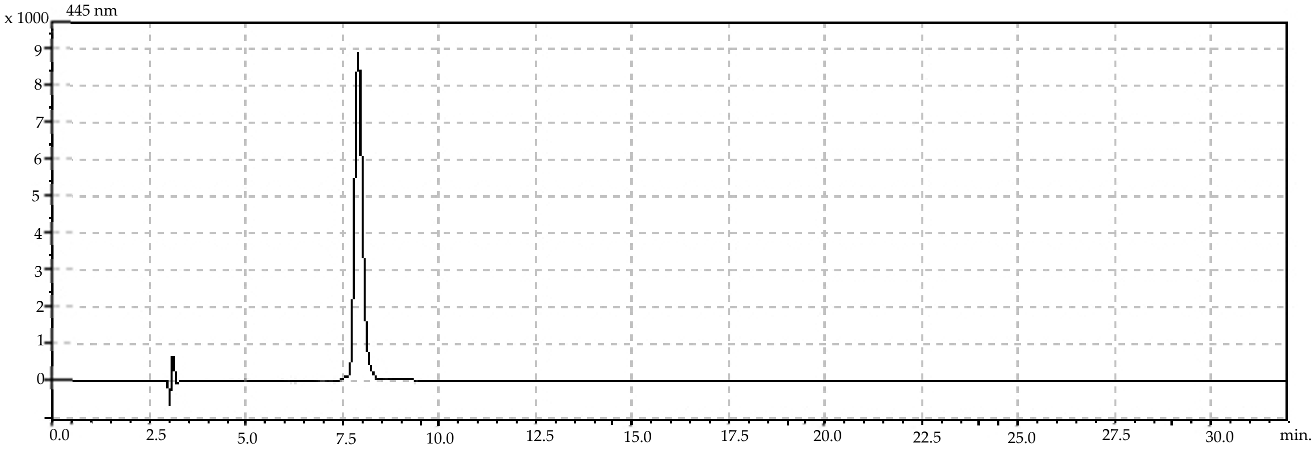

Figure S1A. Pick of pure lutein (standard)

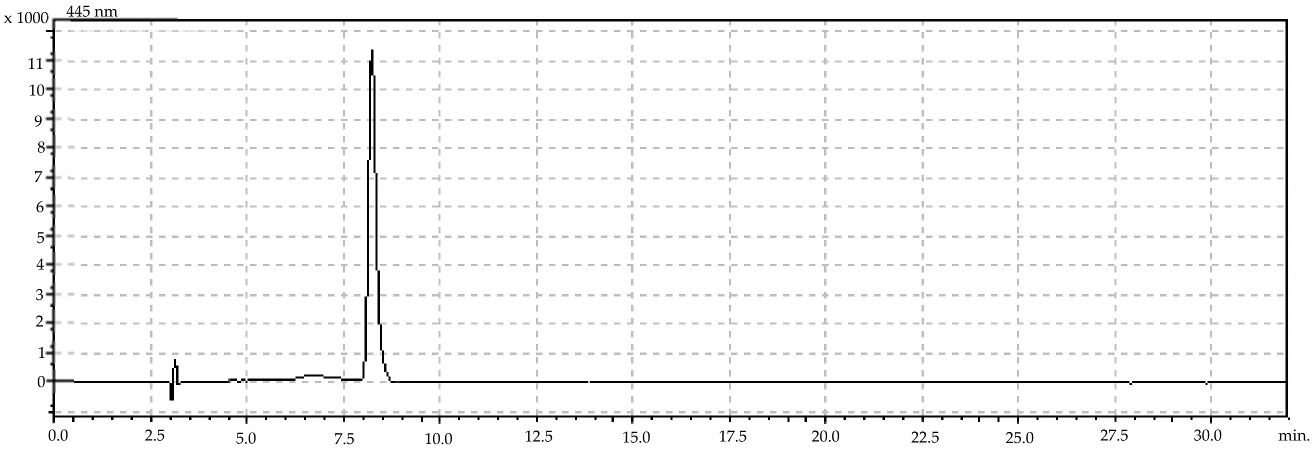

Figure S2A. Pick of pure zeaxanthin (standard)

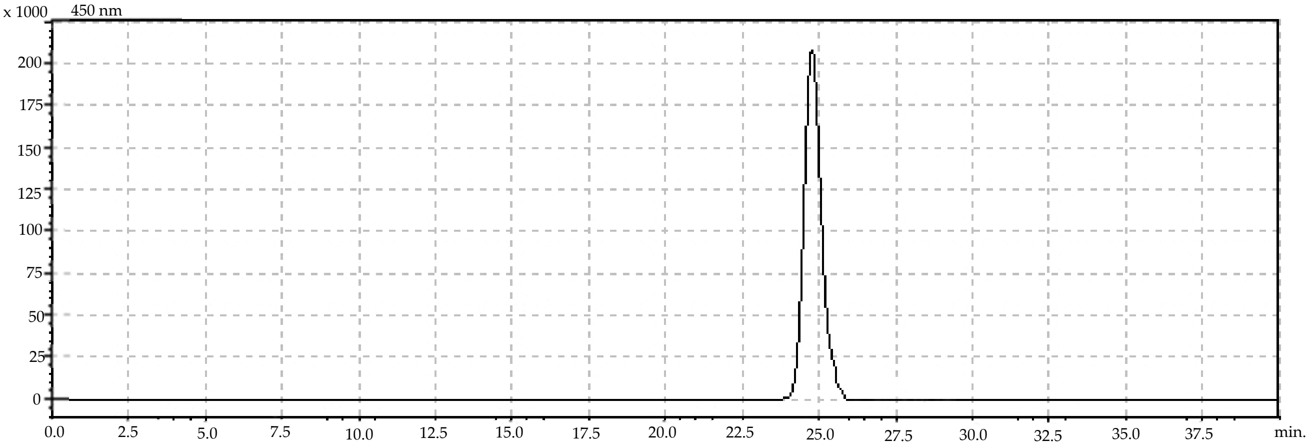

Figure S3A. Pick of pure beta-carotene (standard)

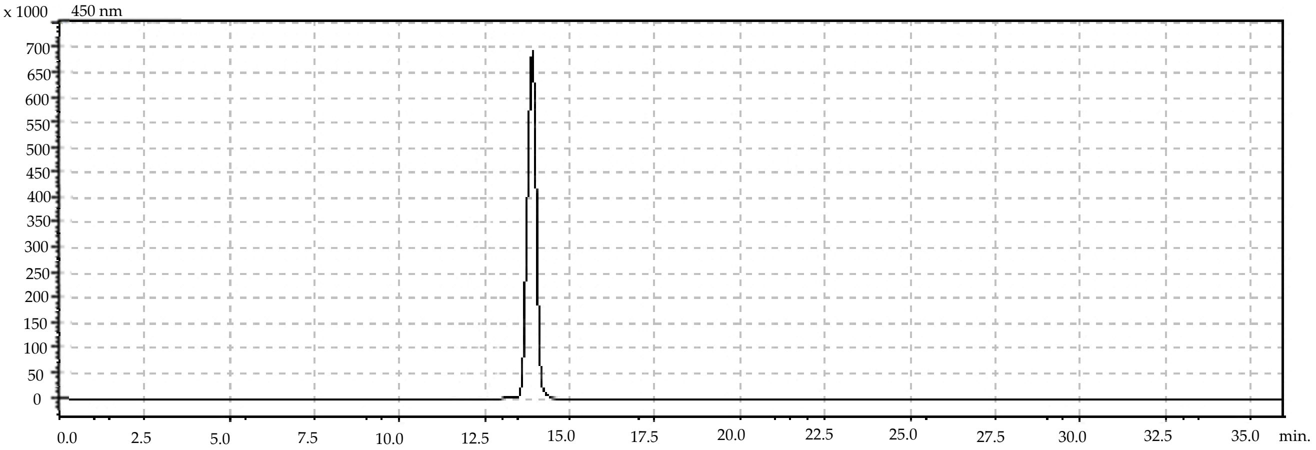

Figure S4A. Pick of pure chlorophyll a (standard)

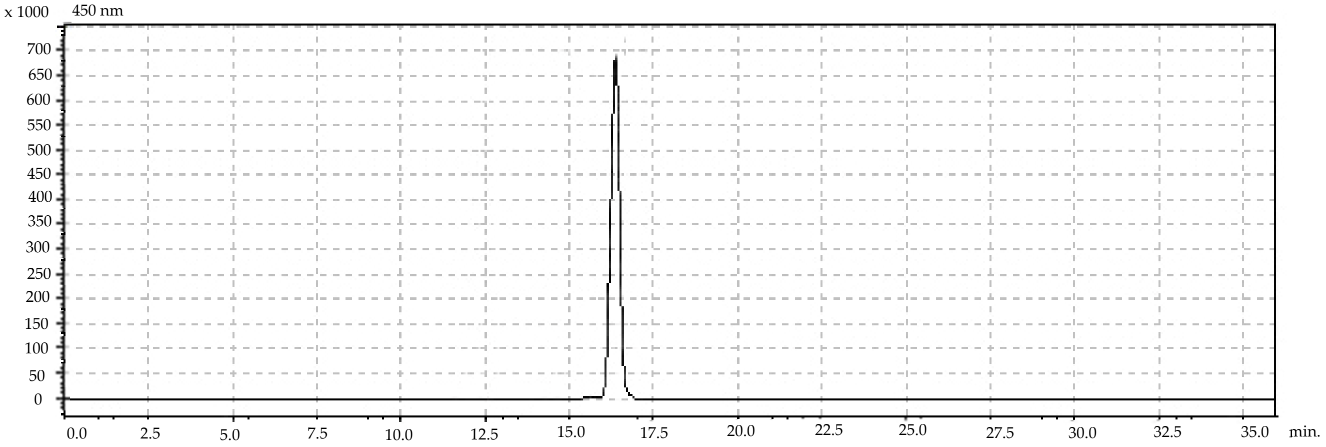

Figure S5A. Pick of pure chlorophyll b (standard)

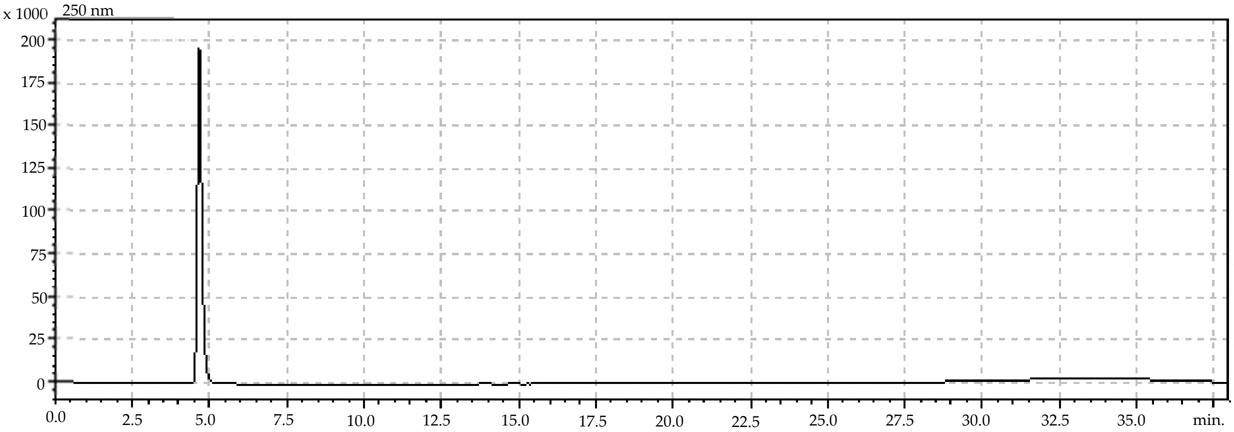

Figure S6A. Pick of pure gallic acid (standard)

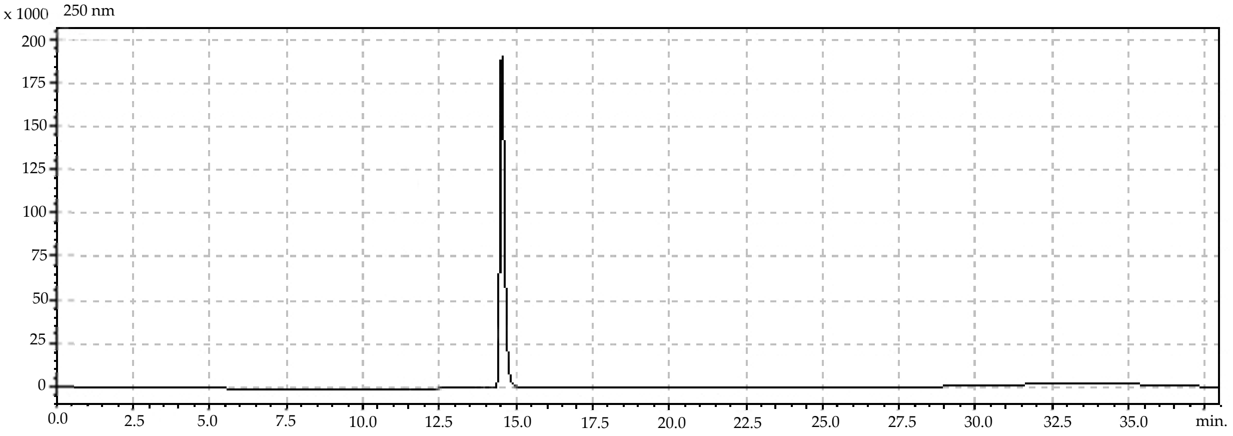

Figure S7A. Pick of pure chlorogenic acid (standard)

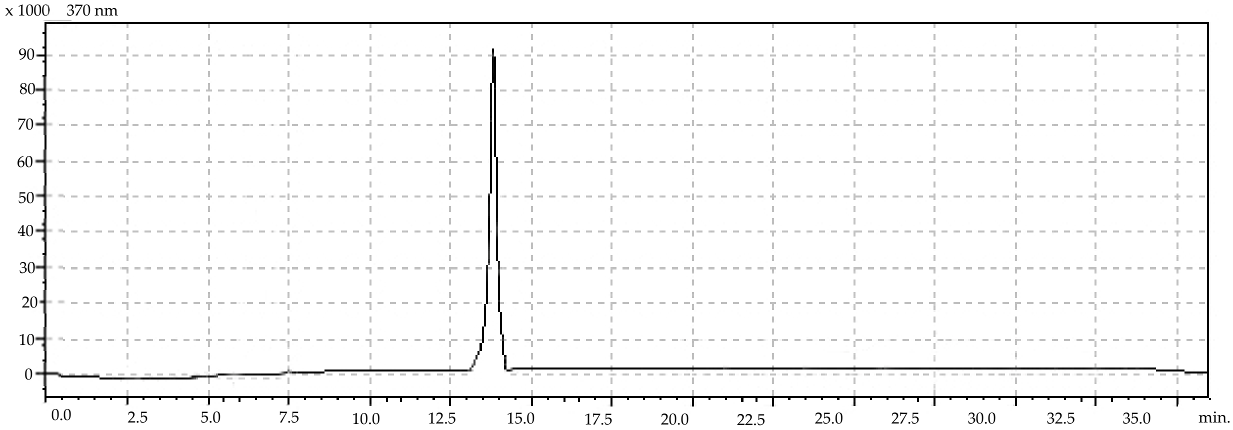

Figure S8A. Pick of pure catechin (standard)

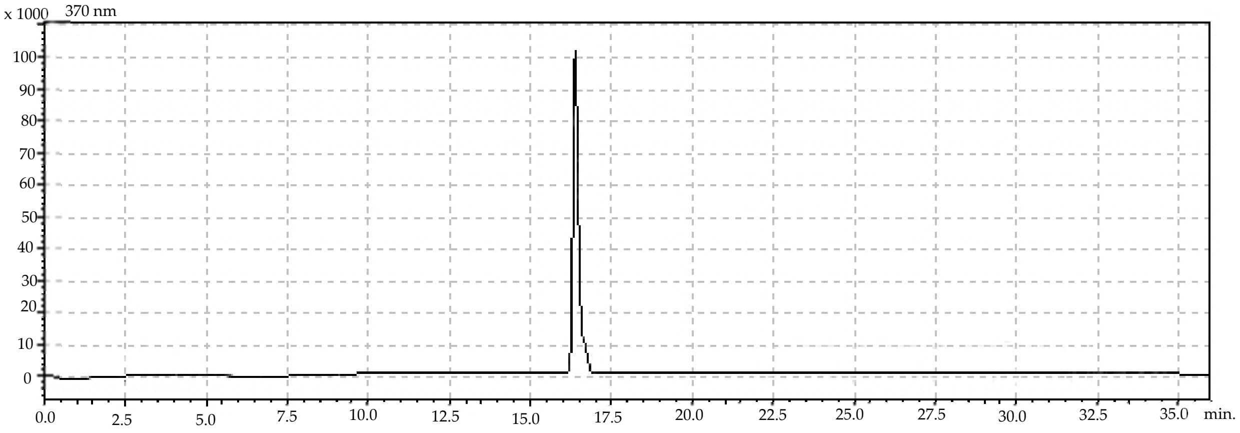

Figure S9A. Pick of pure epigallocatechin (standard)

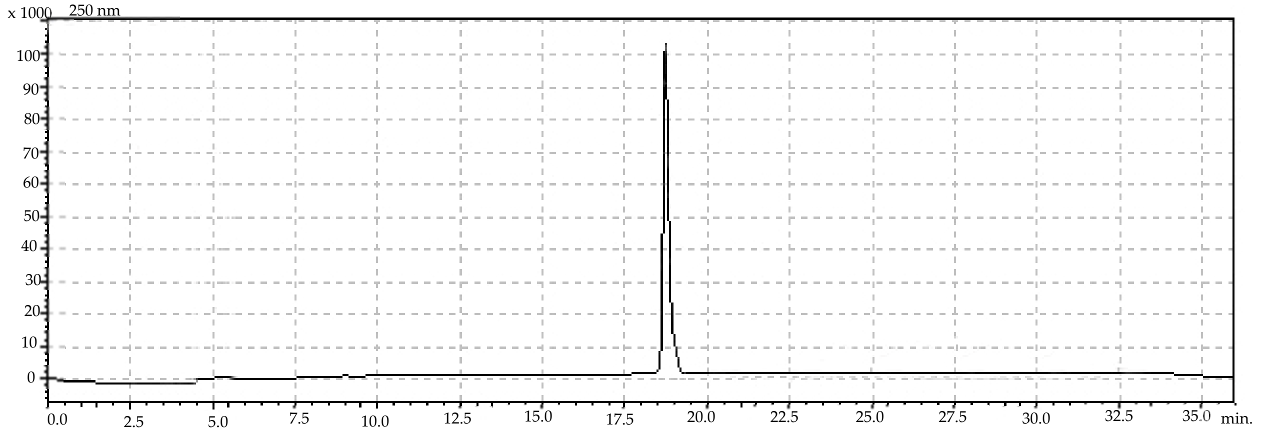

Figure S10A. Pick of pure caffeic acid (standard)

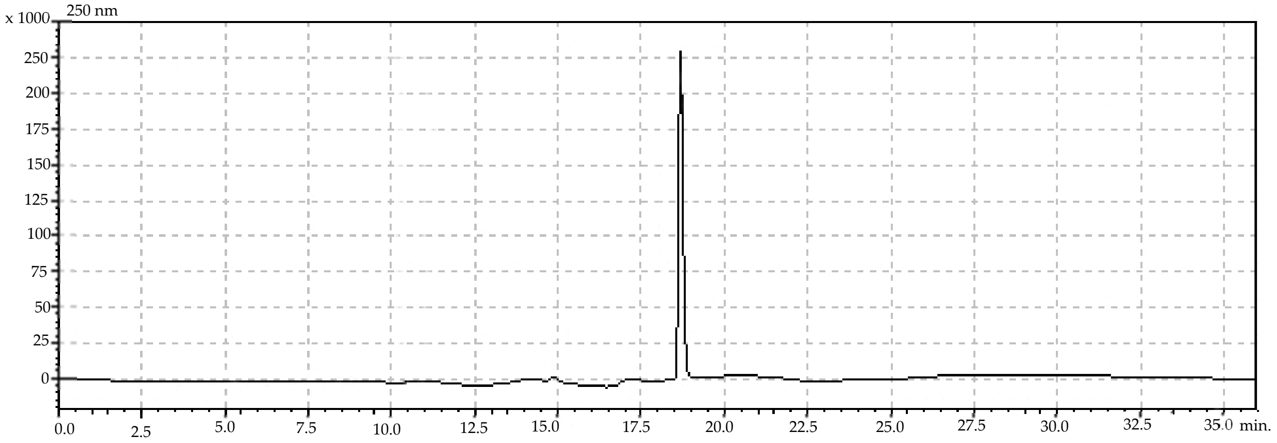

Figure S11A. Pick of pure quercetin-3-O-rutinoside (standard)

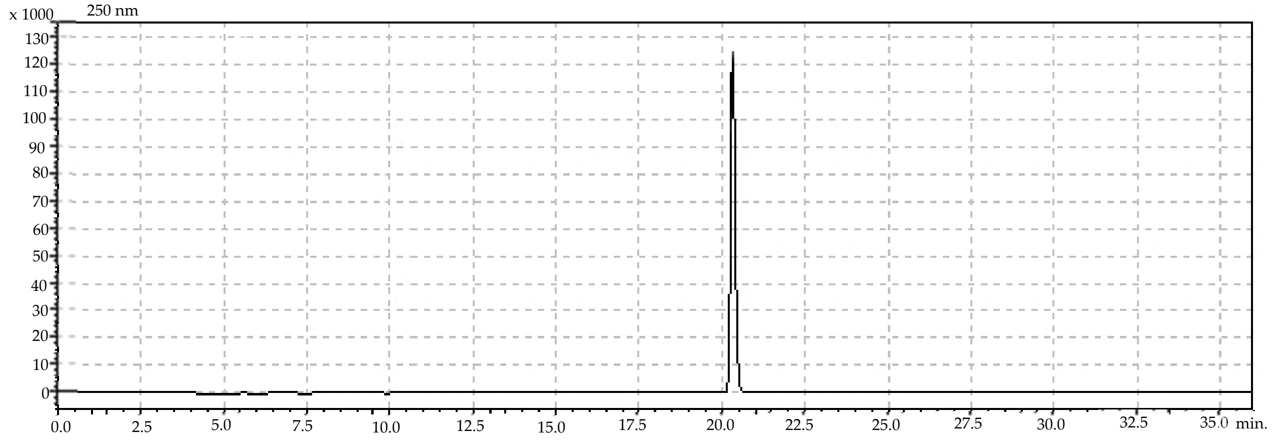

Figure S12A. Pick of pure p-coumaric acid (standard)

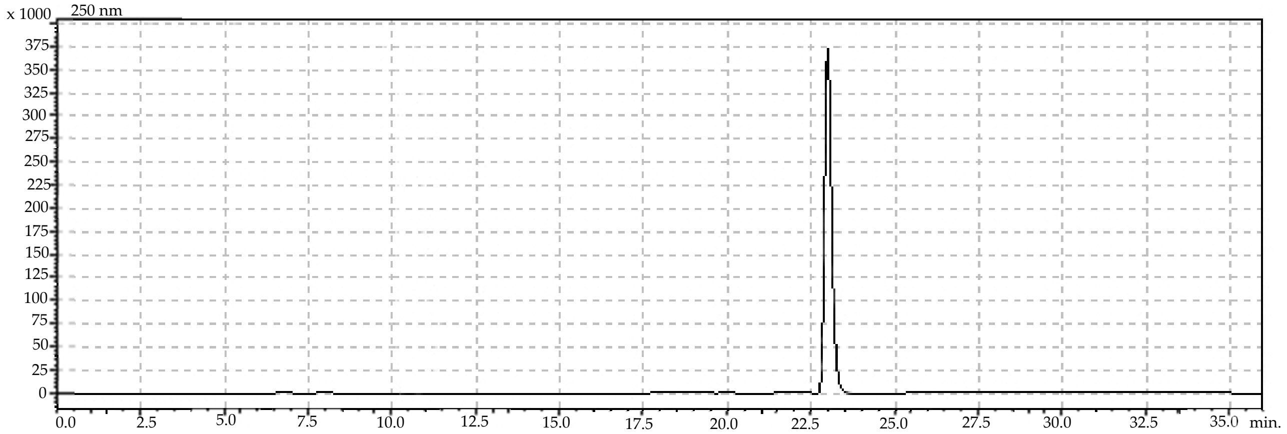

Figure S13A. Pick of pure ferulic acid (standard)

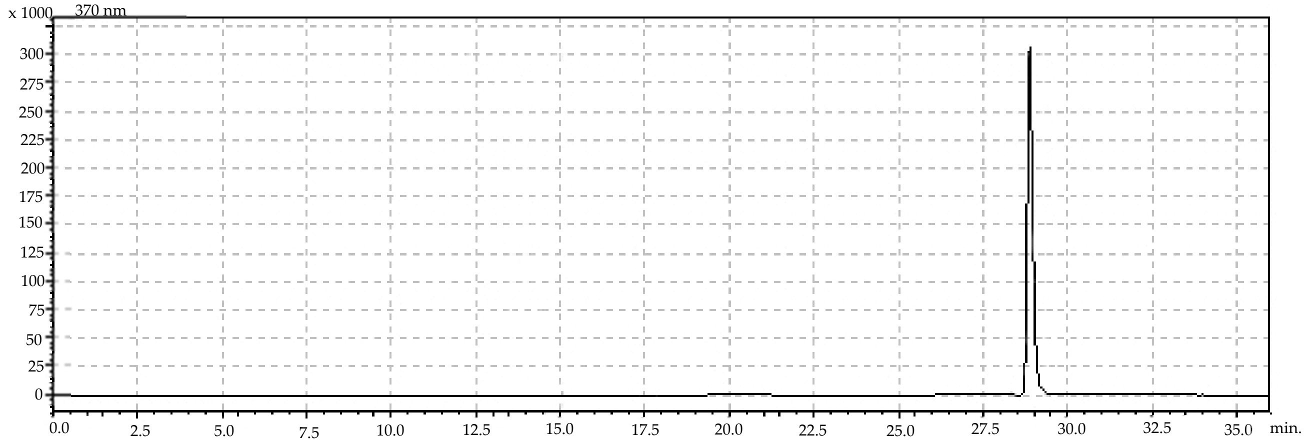

Figure S14A. Pick of pure luteolin (standard)

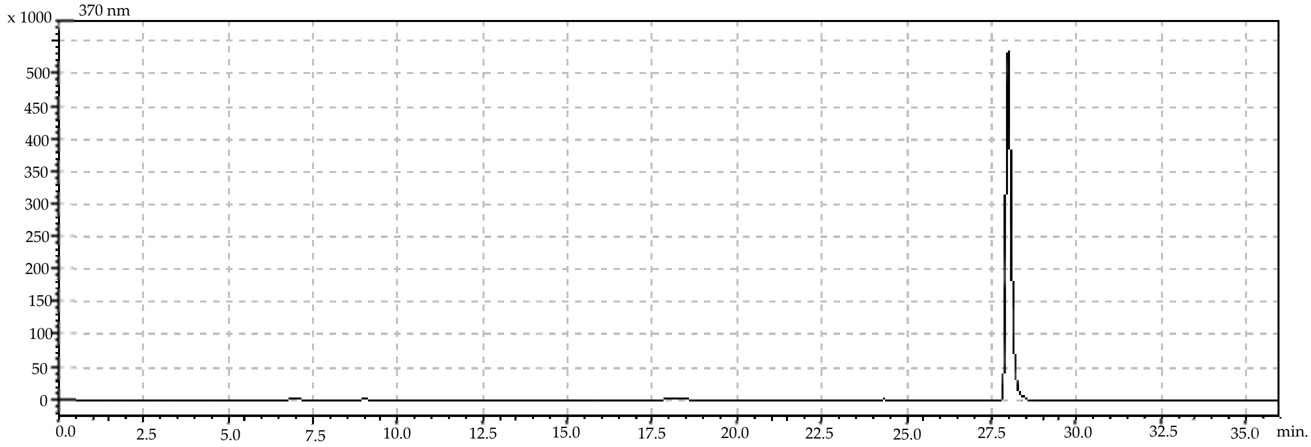

Figure S15A. Pick of pure quercetin (standard)

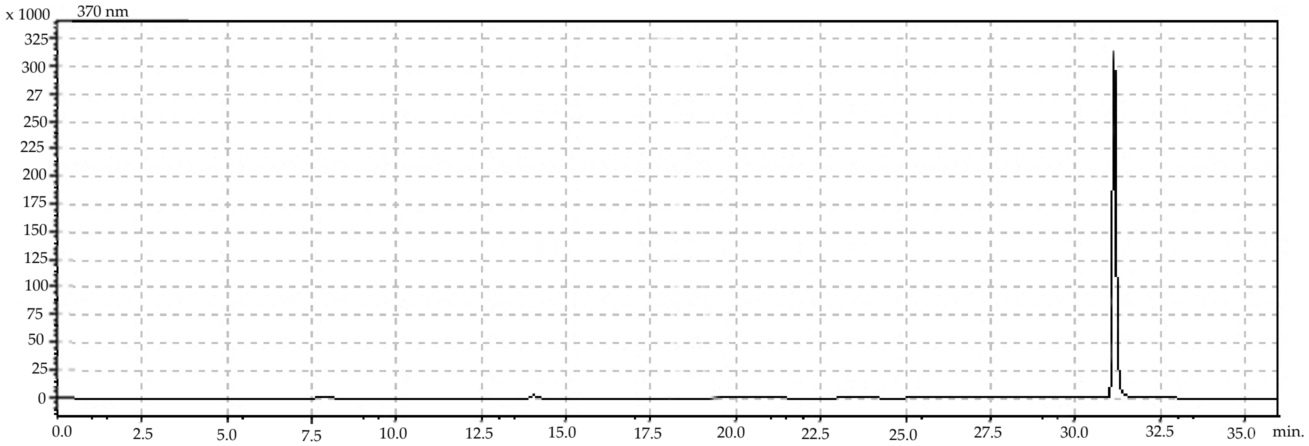

Figure S16A. Pick of pure kaempferol (standard)

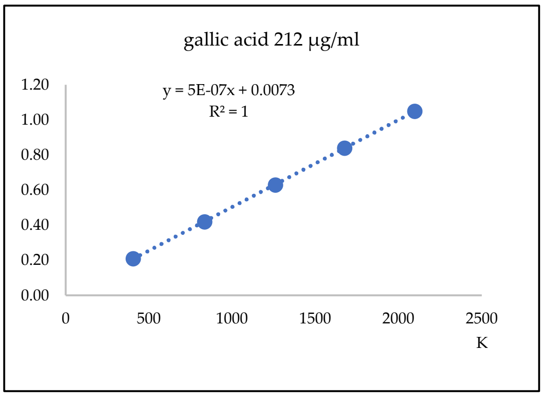

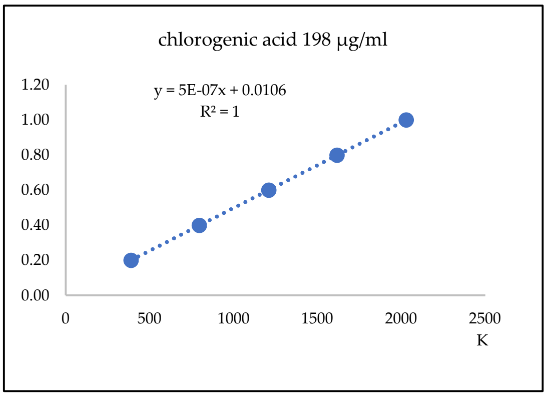

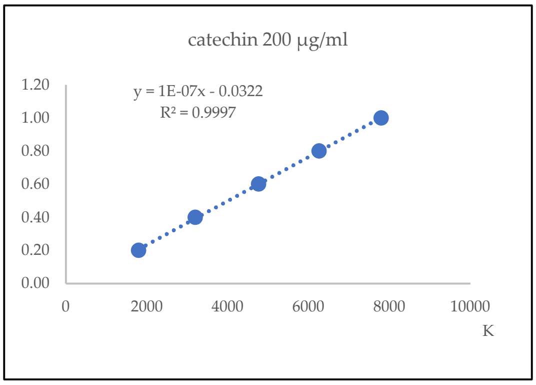

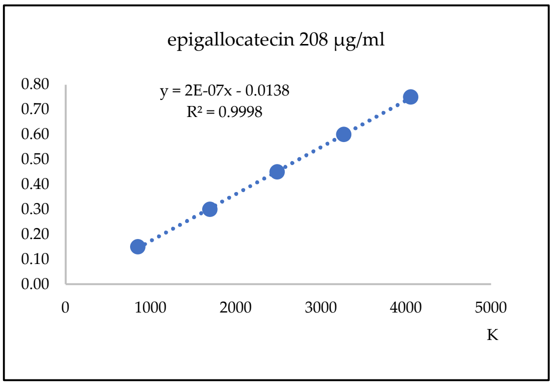

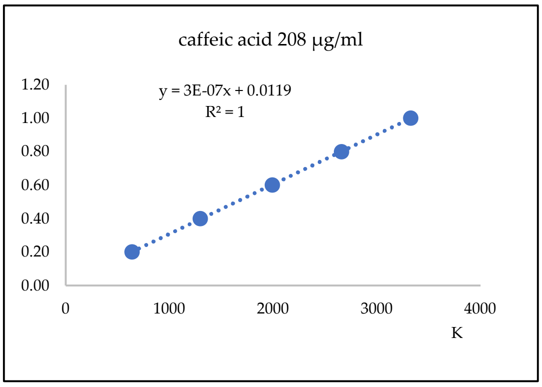

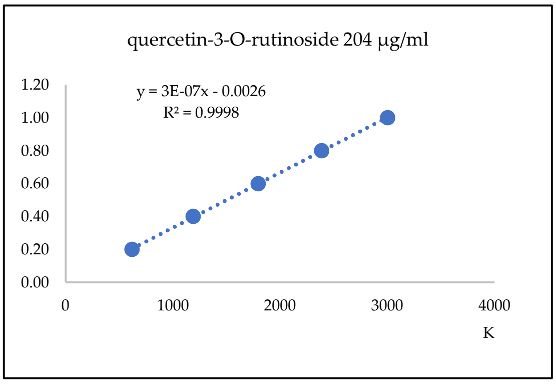

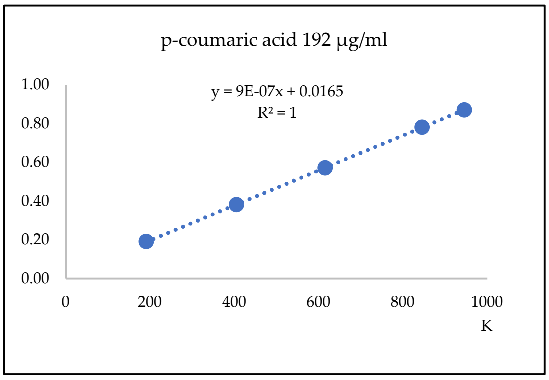

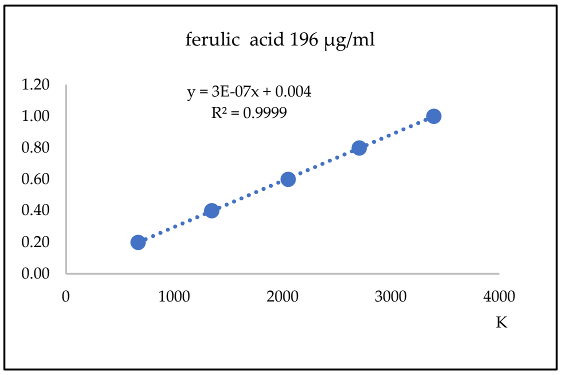

Figure S17A Standard curves for all compounds identified in Chopin apple

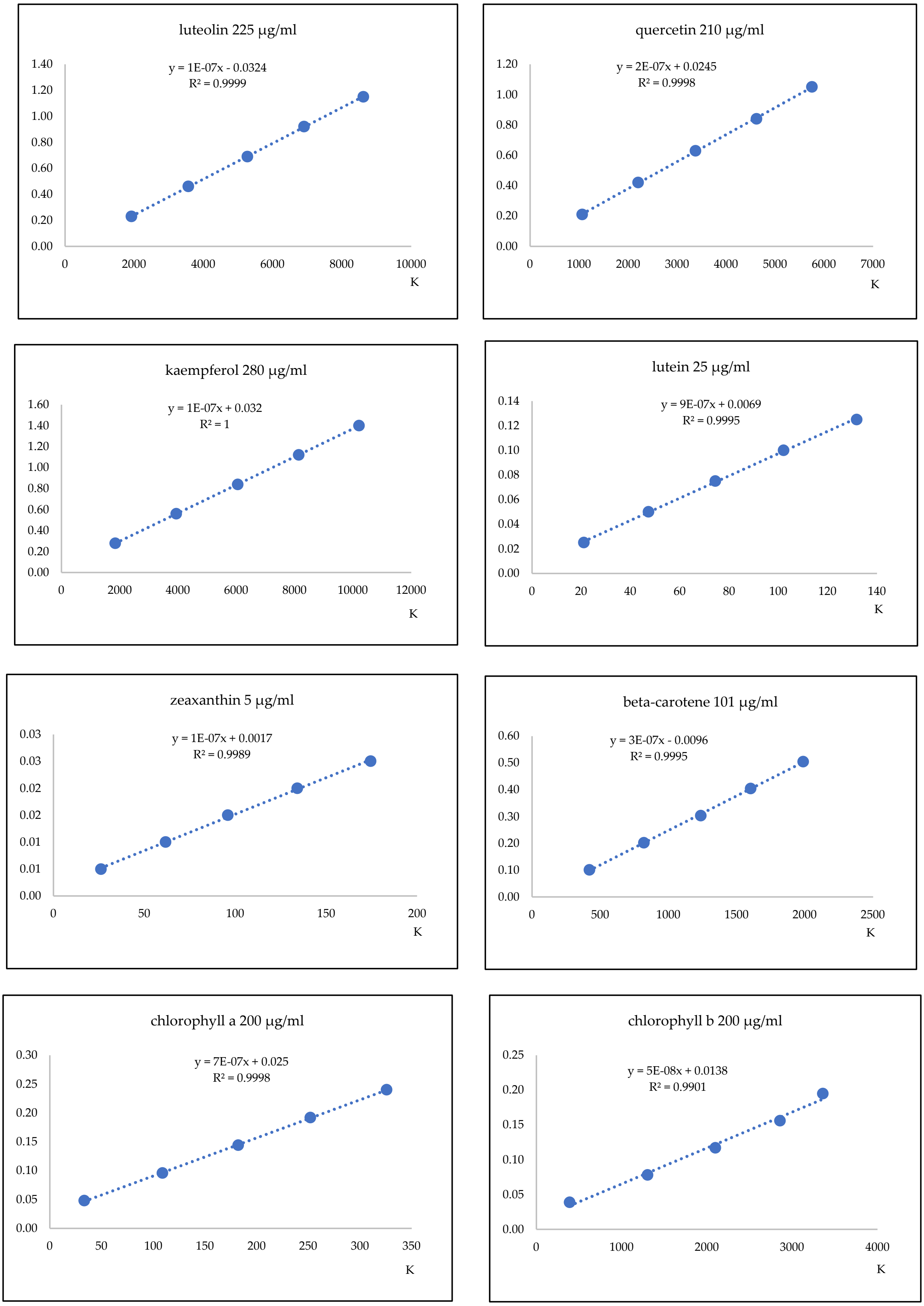

Figure S18A Standard curves for all compounds identified in Chopin apple
